# Plague in Bombay—Haffkine Serum

**Published:** 1899-12

**Authors:** William T. Fee

**Affiliations:** Consul.; Bombay


### Plague in Bombay—Haffkine Serum.

[Advance Sheets of Consular Reports, November 8, 1899.]

BOMBAY, September 7, 1899.

The plague in the Bombay Presidency continues. It had its inception in the latter part of the year 1896, and has spread into every collectorate and district of the Presidency. From statements showing the mortality from plague furnished me by the government of India, I find there were, during the week ended September 3, 1899, 4390 deaths from the plague in the Presidency of Bombay.

Its progress in the southern Maratha country during the past year has been remarkable. It has assumed an epidemic form in the city of Poona, a place of over 100,000 inhabitants, which, on account of its supposed sanitary condition—high elevation, dry heat, with a climate from May to November like an English spring morning—has been made the home for the Bombay army and the residence of the governor during the monsoon season.

This city has had 23,331 cases and 17,809 deaths from plague up to the present time. The epidemic is just at present at its height, and there is great suffering and distress. During the week ending September 2, 1899, there were 1086 deaths from plague, and on Monday, September 4, 1899, 117 cases and 110 deaths were reported in that city.

Western India being now threatened with famine on account of the failure of the monsoons to produce the usual amount of rain to nourish the crops and induce late sowing, it is feared that the coming season will witness a further ravage of the plague.

The distance may save America, yet this scourge repeatedly reached Europe in the early centuries. It is now raging in the East, and there is reason to fear that it is gathering force. It has appeared in Alexandria, Egypt, and Oporto, Portugal, which is only a week's journey from the western world, and the mediæval plague is quite capable of taking advantage of modern rapid traveling.

The plague is an acute infective fever; its primary cause is a living organism, a minute microscopical being which, having gained entrance to the body, multiplies with great rapidity, producing a series of local disturbances giving rise to characteristic symptoms, and diffusing throughout the body a subtle poison which is generally the cause of death.

Our modern physicians are not much better equipped for the treatment of the disease than were their mediæval predecessors. Inoculation, although its results are extremely important and promising, is a prophylactic rather than a treatment, a wall against the enemy rather than a weapon with which to meet it. There are, of course, great advances in the general treatment of the cases, but modern science has not yet discovered a specific against plague.

The plague microbes are capable of living and multiplying only while they have access to supplies of organic nourishment. They are able to pass directly from body to body or to remain alive and even multiply outside of the body. Thus, there are two ways in which plague may travel; it may creep from patient to patient in a direct chain, or it may use places where suitable decaying substances are to be found as temporary links in the chain.

An antitoxine, or serum, first prepared by Professor Haffkine as a plague inoculation, called Haffkine's Prophylactic, is now being used in Bombay and western India with remarkable results.

This prophylactic is prepared by first taking the plague bacilli, or the young germs, from a person affected with the plague and cultivating them. These microbes are killed by artificial means and a high degree of heat. From these dead germs and their poisonous excrements is produced a fluid that is believed to have acquired the power, when injected into the human system, to render the blood immune from the attack of plague germs and to neutralize their effect. The injection of such a poison has the effect of an antitoxine and prevents the system from nourishing plague. A dead plague germ being inoculated into a person, plague will not follow. A person after having one attack of the disease is rarely liable to a second. The person first inoculated is subject to symptoms of the plague. In vaccination for smallpox a living germ is dealt with, whereas in plague inoculation dead seed only are injected.

Experiments are now being carried on at the laboratory at the government house, Parel, Bombay, where further discovery is expected to perfect this plague preventative.

Strangely enough, inoculation is exceedingly unpopular among the natives. The government has had great labor in persuading the Hindoo mind of the efficacy of Haffkine's Prophylactic against plague, and at the same time its utter harmlessness in every other respect.

The Hindoo is suspicious that the dead germs and their toxic excreta may be of animal rather than vegetable substance, which would make the injection of the fluid into their body a religious offense.

The measures generally relied upon in the dry season were entire evacuation of infected villages and hamlets, isolation of the sick, segregation of the "contacts," and a thorough disinfection of all infected places. In localities where the outbreak grew virulent in the monsoon, evacuation was impossible; but inoculation was extensively tried and strikingly demonstrated the protective power of the prophylactic.

At first the people failed utterly to understand the use of segregation. To the masses, infection and contagion had no meaning. Nothing but the splendid confidence of the people in the British government could have made sanitary measures possible, in the broadcast and rapid manner in which it became necessary to use them among untrained and uneducated millions.

Owing to the enormous population of the city of Bombay, and also to the fact that many plague cases are suppressed, secreted or returned under fever or other causes, it is impossible to get general statistics of the effects of Haffkine's plague inoculation. However, I have a few returns from up-country places of the results of inoculation.

At Kirkee, the plague broke out among the royal artillery followers. They were living under far better conditions than many villages, and a cordon had been drawn around that place. Six hundred and seventy-one persons were inoculated and 859 not inoculated. Among those inoculated, there were 32 attacks and 17 deaths, giving a mortality of 2.05 per cent.; while among the uninoculated, there were 143 attacks and 98 deaths, or a mortality of 11.4 per cent.

At Belgaum, among the men of the Twenty-sixth Madras Infantry, after all the Sepoys had been inoculated, there were only two attacks, both of which recovered, whereas before the inoculation, there had been 78 cases in the regiment.

At Ganeshkhind, among the servants of the governor of Bombay, in a certain quarter, there were 324 persons inoculated and 300 uninoculated. Fourteen cases of plague occurred in that quarter, and every one of those cases occurred among the 300 who were not inoculated.

Bacteriological and chemical analyses of the atmosphere and ground, air, etc., are being made in these infected places, results of which may be of extreme interest and infinite use in the future.

WILLIAM T. FEE, Consul.

(From Marine Hospital Reports.)

